# Neglected Hosts of Small Ruminant Morbillivirus

**DOI:** 10.3201/eid2412.180507

**Published:** 2018-12

**Authors:** Claudia Schulz, Christine Fast, Kore Schlottau, Bernd Hoffmann, Martin Beer

**Affiliations:** Friedrich-Loeffler-Institut, Greifswald–Insel Riems, Germany

**Keywords:** Sus scrofa, peste des petits ruminants virus, small ruminant morbillivirus, morbillivirus, transmission, emerging disease, experimental infection, reservoir host, viruses

## Abstract

Eradication of small ruminant morbillivirus (PPRV) is targeted for 2030. PPRV lineage IV is found in much of Asia and Africa. We used PPRV lineage IV strain Kurdistan/2011 in transmission trials to investigate the role of pigs, wild boar, and small ruminants as PPRV reservoirs. Suids were a possible source of infection.

Peste des petits ruminants is one of the most serious (economically and clinically) transboundary animal diseases (*1*–*3*). Of 4 lineages, small ruminant morbillivirus (previously called peste des petits ruminants virus; PPRV) lineage IV (PPRV-LIV) has spread the most widely in the past decade, particularly in Asia, and increasingly dominates the PPRV lineages in Africa (*2*,*4*). Morbidity and mortality rates for goats are high, up to 100%; however, sheep can be subclinically infected and play a major role in the silent spread of PPRV over large distances and across borders (*3*). The role of other wild and domestic Artiodactyls in the epidemiology of PPRV is unknown or insufficiently understood (*3*). Pigs are considered dead-end hosts for PPRV (*5*). In an experimental infection study, pigs infected with PPRV lineage II (LII) strains did not transmit PPRV to goats, but they can transmit the closely related Rinderpest morbillivirus to cattle (*6*). To determine the pathogenesis of PPRV-LIV infection in pigs and wild boar and the capability of these suids to transmit PPRV in comparison with that of goats and sheep, we conducted 4 independent transmission trials during 2015–2016. The experimental protocol was reviewed by a state ethics commission and approved by the State Office for Agriculture, Food Safety and Fisheries of Mecklenburg-Vorpommern, Rostock, Germany (LALLF M-V/TSD/7221.3-1-018/14).

## The Study

 In 4 trials (trials 1–4; Table) we intranasally inoculated suids with a recent PPRV-LIV strain (Kurdistan/2011 [*7*,*8*]). Contact control animals were added 2 days later. We recorded clinical signs and temperature regularly and collected samples to evaluate the suitability of different virologic, serologic, and pathological methods for detecting PPRV infection. We conducted statistical analyses to calculate whether PPRV RNA loads in secretions and excretions (oral, nasal, and fecal swab samples) collected over time from pigs, wild boar, goats, and sheep differed significantly and to determine correlations between the results of virus isolation and PCR assays by using swab samples and purified leukocytes as sample materials (Technical Appendix).

**Figure 1:**
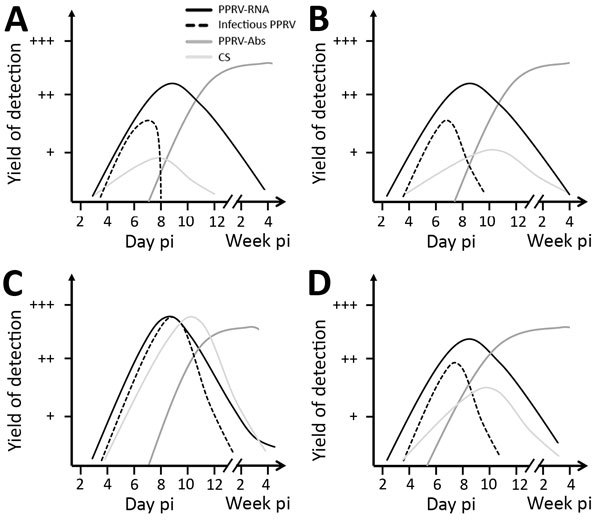
Progression of virologic, serologic, and clinical parameters analyzed in pigs (A), wild boar (B), goats (C), and sheep (D) in Germany after experimental infection with PPRV lineage IV strain Kurdistan/2011. Results are shown for reverse transcription quantitative PCR (solid black lines), endpoint dilution assay (dashed black lines), competitive ELISA (dark gray lines), and clinical score sheets (light gray lines). A detailed description of the infection experiment is provided in the Technical Appendix). Abs, antibodies; CS, clinical signs; pi, postinfection; PPRV, small ruminant morbillivirus (formerly called peste des petits ruminants virus).

**Figure 2:**
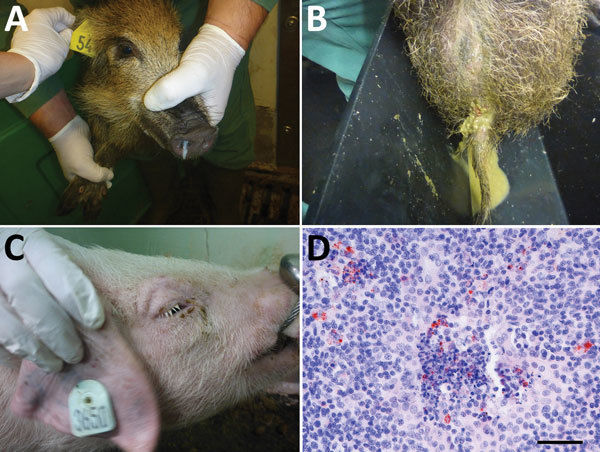
Clinical signs observed in wild boar and pigs and small ruminant morbillivirus (formerly called peste des petits ruminants virus; PPRV) antigen detection in a pig tonsil in experimental study of PPRV transmission, Germany. A) Purulent nasal discharge in wild boar 4 at 8 days after infection; B) diarrhea in wild boar 4 at 7 days after infection; C) swollen eyelids in pig 3 at 10 days after infection; D) PPRV antigen (red) in the tonsil of pig 1 at 30 days after infection (≈22 days after contact infection of pig 1), by immunohistochemical staining with monocloncal mouse anti-PPRV; scale bar indicates 50 μm. Clinical signs in the 3 pigs in trial 1 included a transient rise in body temperature, ruffling bristles, diarrhea, reduced activity and food intake/slight emaciation, swelling of the eyelids, mild to severe conjunctivitis, and mucous to purulent ocular discharge in the first days after infection. In the 4 wild boar in trial 2, clinical signs included transiently increased body temperature, diarrhea (including fresh blood), reduced general condition, inappetence, and mucopurulent nasal discharge. A detailed description of the infection experiments is provided in the Technical Appendix.

As expected, goats showed the typical moderate to severe clinical signs (trials 1 and 3) reported previously (*7*–*9*). Clinical signs in PPRV-infected sheep (trial 4) were generally mild to moderate, as previously described (*3*,*8*). Contact controls showed similar clinical signs. One PPRV-infected sheep showed severe clinical signs similar to those of the goats. Surprisingly, all PPRV-infected pigs and wild boar (trials 1–3) showed various mild to moderate clinical signs, including fever and reduced general condition (all suids), diarrhea (pig 1, boar 1–4), and ocular (pigs 1–3) and nasal (boar 4) discharge typical for PPRV infection (Figure 1; Figure 2, panels A–C; Technical Appendix). PPRV-induced immunosuppression may predispose affected animals to secondary infections (*3*,*9*) as reflected by distinct severe leukocytopenia in pigs and goats a few days after inoculation. Different expressions of clinical signs after PPRV infection might have been caused by concurrent infections with other pathogens or differences in individual resistance to PPRV infection (*9*). In the 4 wild boar, for example, *Balantidium coli*, detected by histopathologic examination (data not shown), might have been an additional factor causing the diarrhea (*10*). Nevertheless, similar to the lack of clinical signs reported for pigs infected with a PPRV-LII strain (*6*), the 2 pigs in trial 3 showed only mild clinical signs.

Contact transmission of PPRV from intranasally infected pigs to 1 contact goat and 1 pig was noted (trial 1). This pig was refractory to intranasal infection but was infected by contact at a similar time as one of the goats. Furthermore, PPRV was transmitted from intranasally infected goats to contact pigs (trial 3) (Table). Hence, in contrast to the findings of Nawathe and Taylor (*6*), who reported contact transmission of a PPRV-LII strain from experimentally infected goats to contact pigs but not vice versa, our transmission trials demonstrated that a complete interspecies transmission cycle of a PPRV-LIV strain between goats and pigs, and possibly also intraspecies transmission between pigs, can be maintained. The virulence of the PPRV lineage or strain is possibly a factor influencing the susceptibility to PPRV infection and the probability of PPRV transmission (*9*,*11*).

**Table:** Design and outcomes of PPRV transmission trials, Germany*

Trial no.†	Trial	No. inoculated animals	No. contact controls	Outcomes			
Seroconversion, total no. by species	Excretion of PPRV RNA, total no. by species	Excretion of infectious PPRV, total no. by species	Contact transmission (no. contact-infected/total no. in contact)
1	P-GP	3P‡	2G, 1P‡	3P,‡ 2G	3P,‡ 2G	1P, 2G	Yes (1/2G;§ 1/1P‡)
2	W-GP	4W	2G, 2P	4W	4W	2W	No (0/2G; 0/2P)
3	G-P	2G	2P	2G, 2P	2G, 2P	2G	Yes (2/2P)
4¶	S-S	5S	5S	5S	5S	5S	No (0/5S)

*P, pig; PPRV, small ruminant morbillivirus (formerly called peste des petits ruminants virus); W, wild boar; G, goat; GP, goats and pigs; S, sheep. †For trials 1–3, animals were experimentally infected by intranasal inoculation with PPRV strain Kurdistan/2011 for independent transmission trials conducted in the containment facility of the Friedrich-Loeffler-Institut, Isle of Riems, Germany. Contact control animals were added 2 d after experimental infection. In 2 of the trials, PPRV transmission was documented from pigs to 1 goat and 1 pig (trial 1) and from goats to 2 pigs (trial 3). Infectious PPRV excretion was detected in >1 animal of each species, and PPRV RNA and seroconversion were detected in all experimentally infected or contact-infected animals (further details in Technical Appendix Figure 1). For trial 4, a 1-to-1 (pairwise) study design was chosen to estimate the reproductive ratio. The results of the sheep trial are presented in this study to enable comprehensive comparison with the PPRV pathogenesis in suids. ‡One of 3 pigs was probably not infected by experimental intranasal PPRV inoculation but by contact infection.  §One contact goat was infected by pigs; however the source of infection (goat or pig) cannot be determined for the second contact goat. ¶In each of 5 stables, 2 sheep were kept together: 1 experimentally infected sheep and 1 contact control sheep.

From 2 of 4 wild boar (trial 2), PPRV was isolated from a few fecal swab samples but was not transmitted to the contact goats or pigs. Unexpectedly, none of the intranasally infected sheep transmitted PPRV to any of the contact sheep. The considerable differences in transmission efficiency between goats and the other Artiodactyls can be explained by higher PPRV loads excreted by goats (Figure 1). Statistically significantly higher PPRV RNA loads over time were found in PPRV-infected goats than in suids and sheep. Peak viral loads in goat samples were up to 1 log step (PCR) and 2.5 log steps (virus isolation) higher (9.3 × 10^7^ copies/mL; 10^6.0^ TCID_50_ [50% tissue culture infective dose]/mL) than in pig and wild boar samples (1.5 × 10^7^ copies/mL; 10^3.5^ TCID_50_/mL). Of note, peak viral loads in sheep (10^4^TCID_50_/mL) were only slightly higher than those in pigs and wild boar, which may explain why none of the sheep transmitted PPRV to the contact control sheep. The higher viral loads in goats could also explain the ≈4 days earlier contact infection of the contact pigs in trial 3 than the contact goat and pig in trial 1. Besides a higher innate susceptibility to PPRV infection observed for goats compared with sheep and suids, the infective dose may play a role in the efficiency of transmission and infection dynamics of PPRV in suids as previously reported for goats (*9*) and camelids (*12*).

We detected seroconversion in all PPRV-infected animals by using competitive ELISA and neutralization tests. Neutralizing antibody titers were moderate to high in suids and goats (2.16–2.96 log_10_ ND_50_ [virus neutralization in 50% of replicates]) and slightly lower in sheep (1.76–2.56 log_10_ ND_50_). After seroconversion, no PPRV could be isolated from swab and purified leukocyte samples, but PPRV RNA was detected in swab samples for at least 3–4 weeks after infection in all species, with individual differences (Figure 1; Technical Appendix). Correlation analyses revealed a poor to excellent correlation of PCR and virus isolation results before seroconversion, depending on the animal species. Possible reasons for (transient) PPRV RNA persistence are infection of neurons followed by transsynaptic spread (*13*). Indeed, PPRV RNA was detected in single or multiple brain samples of 2 sheep, 4 goats, and in the choroid plexus of 1 pig, 1 wild boar, and 3 goats. PPRV RNA in the choroid plexus might have been associated with PPRV-infected immune cells, as has been reported for ferrets infected with closely related canine morbillivirus (*14*). Immunohistochemistry demonstrated that PPRV antigens in other tissues were often associated with immune cells. For PPRV diagnosis in the examined species, tissue of the lymphoreticular system, in particular tonsils (Figure 2, panel D), head and lung–associated lymph nodes, mesenteric lymph nodes, and small intestinal Peyer’s patches, were found most suitable for postmortem diagnosis with PCR and immunohistochemistry. PCR was the most sensitive virologic method independent from the sample material, and competitive ELISA proved reliable for serologic PPRV diagnosis (online Technical Appendix).

## Conclusions

Transmission trials with a virulent PPRV-LIV strain revealed that suids are an unexpected possible source for PPRV infection. Therefore, domestic pigs and wild boar should be considered as possible PPRV reservoir hosts. This finding is especially relevant to stringent control programs. The epidemiologic role of suids in the spread of PPRV, as maintenance or spillover hosts (*15*), should be further investigated in field and experimental studies using different PPRV lineages and strains at different environmental and experimental conditions.

Technical Appendix Additional methods and results for study of neglected hosts of small ruminant morbillivirus.
