# Supplementary material for: Neglected Hosts of Small Ruminant Morbillivirus
Source: Emerg Infect Dis. 2018 Dec;24(12):2334–7. doi: 10.3201/eid2412.180507 (PMC6256395; doi:10.3201/eid2412.180507)
Supplement: Technical Appendix — Additional methods and results for study of neglected hosts of small ruminant morbillivirus. [file 18-0507-Techapp-s1.pdf]

Article DOI: https://doi.org/10.3201/eid2412.180507

Neglected Hosts of Small Ruminant Morbillivirus

Technical Appendix

Material and Methods

Virus

For experimental infection, a recent isolate of the small ruminant morbillivirus lineage IV strain peste-des-petits-ruminant virus (PPRV) Kurdistan/2011 (lab submission no. BH15/11–5; Accession no. JF969755.1, KF648288, KF648287.1) was obtained from a lung sample from a wild goat (bezoar ibex, *Capra aegagrus*) in Iraqi Kurdistan (*1*,*2*). Briefly, after initial isolation on CHS-20 (goat-SLAM) cells (*3*) the virus was passaged twice on vero.dog.slam.tag cells (*4*) (10^4.83 $TCID_{50}$/ml, quantification cycle (Cq) value 15.01). This virus stock (PPRV Kurdistan/2011/BH15–11_5/1CHS/2VDS 11/06/14) was used for all animal trials in this study.

Animals and study design

To investigate the role of the 4 different *Artiodactyla* species pig, wild boar, goat, and sheep, as potential reservoirs for PPRV, 4 independent transmission trials were conducted. The experimental set-ups, trial and animal numbers (no.) and IDs are given in the Table in the main text and in Technical Appendix Table 1. All animals were housed in the containment facility of the Friedrich-Loeffler-Institut, Isle of Riems, Germany. Before experimental PPRV-infection, all animals were healthy, dewormed and tested serologically and virologically free of a previous PPRV infection. Three pigs, 4 wild boar, 2 goats and 5 sheep were intranasally (i.n.) infected with 2 ml of ≈$10^{4.5}$ $TCID_{50}$/ml (1ml in each nostril) using nasal atomizers (LMA MAD 100, Wolfram Droh GmbH, Mainz, Germany).

Two days after experimental infection (dpi), PPRV-naïve contact-control animals were added. In trials 1 to 3, goats (highly susceptible to PPRV-infection) were added as contact control animals. In trial 2, two pigs were additionally kept as contact animals in the same compartment than the four wild boar. In trial 1, one pig served as a contact control for pigs since

this pig was refractory to experimental PPRV-infection. The goats of trials no. 1 and 2 had to be kept separately from the pigs or wild boar to avoid injuries due to aggressive behavior against animals of the other species. Animals were separated by a fence but kept in direct contact. Goats and suids of trials 1 and 2 were kept in the same compartment in direct contact under supervision during the time of cleaning and sampling (around 2 hours/day). The goats and pigs of trial 1 exchanged their compartment section daily. Therefore, in a daily rotation system, goats were kept in the compartment section where pigs were kept the day before, without cleaning procedure, and vice versa, the pigs were kept in the former compartment section of the goats, but after cleaning of the section. In trial 3, the two goats and two pigs were kept in the same compartment from 2 dpi after the infection of the two goats until the end of the animal trial (21 dpi). The sheep trial (no. 4) was primarily designed to estimate the reproduction ratio (R_0) using a one-to-one ("pair-wise") study design. The results of the sheep trial are presented in this study to allow a comprehensive comparison of the PPRV pathogenesis in 8 different *Artiodactyla* species.

Rectal body temperature was recorded daily from a few days before experimental infection until the end of the experiments. Clinical signs were evaluated using the clinical score sheet published by Pope et al. (*5*) for small ruminants. For the evaluation of clinical signs in suids, the clinical score sheet of Pope et al. (*5*) was adapted to clinical parameters published for pigs (*6*–*8*) (see Technical Appendix Table 2). The clinical score of all animals was assessed by the same veterinarian. Oronasal, conjunctival and fecal swab samples as well as serum and EDTA-treated whole-blood were collected at regular intervals (Technical Appendix Figure 1). Urine samples were collected from pigs (P1 to P3) at 8, 10, and 14 dpi and at post-mortem examination at 16 dpi (P2) or 30 dpi (P1 and P3) and from goats at post-mortem examination at 19 dpi (G5) or 30 dpi (G4) (Technical Appendix Table 3). The swab samples were collected using dry cotton swabs (Ø 4mm, AMEFA GmbH, Limburg, Germany) and directly soaked and incubated in 2 ml of minimal essential medium (MEM) with 5% fetal bovine serum (FBS), antibiotics and antimycotic. After shaking for 30 min at room temperature, samples were directly titrated before freezing or directly frozen at –80°C until analysis (see below).

Serology

Serum samples of all animals were analyzed with competitive ELISA (ID Screen® PPR competition, ID.vet, France) to detect antibodies against PPRV Np at multiple dpi (Technical

Appendix Figure 1). Selected samples taken before experimental PPRV-infection and in the end of the animal trials were additionally tested in a standard microneutralization test against the virus isolate used for experimental infection. The antibody titer (virus neutralization in 50% of the replicates, ND_{50}) for each serum was calculated according to Spearman and Kärber (*9*). Neutralizing antibody titers <1.5, 1.5 to 2.5 and >2.5 $log_{10}ND_{50}$ were considered low, moderate and high, respectively.

Leukocyte purification and counting

For the purification of viable leukocytes from whole blood, erythrocytes were lysed using ammonium chloride and leukocytes were purified similar to Dagur and McCoy (10), resuspended in ZB 18 medium (Bio Bank, FLI, Insel Riems, Germany) containing 15,9 g/L RPMI 1640 with Hepes 25mM (Gibco), 2 g/L NaHCO3 (Roth), 2.5 g/l Glucose (Sigma), 0.12 g/L Na-Pyruvat (Merck), 0.073 ml/L (0.004 mM) of 55mM 2-Mercapthoethanol (Gibco), 10% FBS, filled up with ultrapure water to 1 L. Resuspended leukocytes were counted in a Neubauer Counting Chamber. Infectivity in leukocytes is given as $Log_{10}[TCID_{50}/10^6$ cells] (Technical Appendix Figure 1).

For the total leukocyte count (cells/ml), erythrocytes in whole blood were lysed by diluting whole blood 1:100 dilution in 3% acetic acid and counting cells in a Neubauer Counting Chamber.

PPRV-RNA extraction and detection

PPRV-RNA from swab, serum, whole-blood, purified leukocytes and homogenized tissue samples was extracted together with an internal control (*11*) using NucleoMagVET kit (Macherey-Nagel, Düren, Germany) at a KingFisher platform (KingFisher Flex, Thermo Fisher Scientific, Schwerte, Germany) as previously described (*12*,*13*). For the detection of PPRV-RNA by real-time quantitative reverse transcription PCR (RT-qPCR), SensiFast Probe No-ROX kit (Bioline) was used with PCR-assays specifically detecting partial PPRV N gene sequences (*14*,*15*) at a Bio-Rad CFX96 real-time system (Bio-Rad Laboratories GmbH, Munich, Germany). Samples from animals of trial 1 were initially analyzed using the PCR-assay of Bao et al. (*15*). Since the analytical sensitivity of the PCR-assay of Batten et al. (*14*) (100 copies/ml) was found slightly higher compared to the Bao PPRV-PCR assay (1000 copies/ml) (data not shown), animal samples of all trials were tested with the Batten PPRV-PCR assay.

Virus isolation

PPRV isolation and titration was conducted with Vero.dog.slam.tag (green monkey) (VDS) cells (*4*) (trials no. 1 to 4) and CHS-20 (monkey CV1) (CHS-20) cells (*3*) (trials no. 2 to 4) that express the signaling lymphocyte activation molecule (SLAM) (CD150) of dogs (*4*) and goats (*3*), respectively. The samples titrated on both cell lines originated from the same dilution series and were titrated at the same time.

Virus isolation was generally conducted from samples with Cq values <34 (sheep; but including all PCR-positive samples collected 4 to 10 dpi), <32 (wild boar; as detected with Batten PPRV-PCR assay) or ≤32 (pigs; as detected with Bao PPRV-PCR assay). Inocula (used for experimental infection) and animal samples were titrated with endpoint dilution assay in Dulbecco's Modified Eagle's Medium (DMEM) with 7.5% to 10% FBS, antibiotics and antimycotic using a ten-fold dilution series - starting with the 1:10 dilution. After adding VDS or CHS-20 cells, titration assays were incubated for 5 to 7 days at 37°C and 5% CO_2. Cells were regularly examined for syncytia caused by cytopathic effect (CPE) to determine 50% tissue culture infective dose ($TCID_{50}$/ml) for the samples.

Indirect immunofluorescence assay

The results of the titration assays were confirmed by immunofluorescence staining together with a positive control (PPRV Kurdistan/2011/BH15–11_5/1CHS/2VDS_PC 15/09/14) of the same PPRV isolate used for experimental infection of animals using anti-PPRV-nucleoprotein (Np) purified monoclonal mouse antibody (Mab anti-PPR, concentration 1 mg/ml, 50% Glycerin, ID.vet) and Alexa 488 (rabbit anti-mouse) fluorophore (Invitrogen, purchased from Fisher Scientific). Therefore, supernatant in the microtiter plate was discarded, cells were washed once with phosphate buffered saline (PBS^-), fixed for 10 minutes with equal amounts of ice cold Aceton and Methanol at 4°C. After discarding Aceton/Methanol, plates were washed once with phosphate buffered saline (PBS^-). The Mab anti-PPR antibody was diluted 1:1000 in PBS^- with 0.05% Tween 20 (PBS-T) and 50 µl of the solution was added to each well. Plates were incubated for 45 minutes at 37°C and 5% CO_2 and then washed twice with PBS-T. For immunofluorescence staining, the Alexa 488 fluorophore was diluted 1:1000 in PBS-T and 50µl of the solution was added to each well. Plates were again incubated for 45 minutes at 37°C and 5% CO_2 and wells washed twice with PBS-T. For immunofluorescence analysis, 50 or 100 µl of PBS^- were added to each well.

Pathology

Selected animals were examined post-mortem for gross pathological lesions. Tissue samples of up to 31 organs were processed for RT-qPCR (Technical Appendix Table 3). Therefore, a piece of tissue of the size of a grain of rice was collected. Of all hollow organs, tissue samples were taken from the inner side to target the mucosa according to (*5*). From tonsils and lymph nodes a cross-section of the different areas was chopped. The tissue pieces were homogenized in 500µl of minimal essential medium (MEM) with a 5 mm steal bead at a TissueLyser (Qiagen) at 30 Hz for 2 min. The tissue homogenates were centrifuged at full speed for 1 min with a table centrifuge and subsequently subjected to homogenization, total RNA extraction and PPRV-RNA detection or additionally to virus isolation and indirect immunofluorescence assays (see earlier).

For histopathological (HP) and immunohistochemical (IHC) examination, tissue samples were fixed in 10% buffered formalin (4% solution of formaldehyde) and embedded in paraffin. Subsequently 3 µm sections were cut, deparaffinised and rehydrated. One section was stained with hematoxylin/eosin, successive sections were used for IHC. Pretreatment included a blocking step for the endogenous peroxidase using 3% H2O2/distilled water followed by an antigen retrieval step using high temperature in citrate buffer (pH 6.0 in microwave). As a primary antibody, the Mab anti-PPR antibody (see earlier) was used in a dilution of 1:100 in Tris-buffered saline (TBS). Negative control sections were treated with TBS alone. The slides were finally developed with biotinylated anti-mouse immunoglobulin and an avidin/biotinylated enzyme complex (VECTASTAIN®ABC Reagent) followed by a visualization with AEC substrate, counterstained with hematoxylin.

Comparison of performance characteristics of different virological methods

Four of the following different virological diagnostic methods were compared for their performance characteristic to detect a PPRV infection in various animal samples (n = 54, whole blood, serum, swabs, tissue, urine) of 4 different *Artiodactyla* species (Technical Appendix Table 4): RT-qPCR and virus isolation (see earlier) as well as antigen-capture ELISA (ag-ELISA) and lateral flow device (LFD). PPRV RT-qPCR assays (comparing primer-probe assays described by Bao et al. (*15*), Batten et al. (*14*) and Polci et al. (*16*)) as well as virus isolation and immunofluorescence were conducted as described herein in the respective sections. The ag-ELISA (ID Screen® PPR Antigen Capture sandwich ELISA, ID.vet) and LFD (PESTE-TEST,

Field test for Peste des Petits Ruminants Virus Infection, BDSL IRVINE LIMITED and The Pirbright Institute, Pirbright, UK) were conducted according to manufacturers' instructions. For the method comparison, only samples positive with Bao and or Batten PPRV-PCR assay were included in this evaluation. To allow a comprehensive comparison of samples containing infectious PPRV, primarily samples positive by virus titration assay were chosen. The analyses of animal samples collected during the trials revealed that RT-qPCR is the more sensitive virological method compared to virus isolation (see also Technical Appendix Table 4). As described earlier, the used RT-qPCR assays showed a high sensitivity of 100 to 1000 copies/ml. Hence, samples positive by virus isolation and/or RT-qPCR were chosen for the comparison of performance characteristics with the other two virological methods (LFD and ag-ELISA).

Statistical analyses

To determine, whether the PPRV-RNA load in excretions collected from animals over time after experimental or contact infection with PPRV lineage IV strain 'Kurdistan 2011' differ statistically significantly by *Artiodactyla* species, Cq values (obtained with PPRV-N-gene-specific RT-qPCR as determined with the PCR assay of Batten et al. (*14*)) of oronasal, conjunctival and fecal swab samples were statistically analyzed. Therefore, the PCR results of oronasal, conjunctival and fecal swab samples collected from PPRV-infected goats (n = 4), pigs (n = 5), wild boars (n = 4) and sheep (n = 5) were included (Technical Appendix Figure 2, Table 3). For contact infected animals, the day of contact infection with PPRV was estimated by comparison of serologic and virological results of experimentally infected and contact infected animals. Accordingly, the day of contact infection (dpci) after i.n. PPRV infection of the experimentally infected animals was estimated 8 dpi for P1, 4 dpi for P5 and P6, 16 dpi for G4 and 8 dpi for G5 (Technical Appendix Figure 1) and consequently assigned as 0 dpci for statistical analyses and visualization in (Technical Appendix Figure 2 and Table 3). The goodness of fit of Cq values of the swab samples collected over time from the different *Artiodactyla* species was tested with Shapiro-Wilk normality test using R software package (*17*), which revealed no normal distribution of these data. Hence, *p*-values were calculated using i) a linear mixed-effects model (lme) including random effects (individual animal) and fixed effects (animal species and dpi as continuous variables) and ii) independent 2-group Mann-Whitney test with Bonferroni correction to adjust the α-level for multiple comparison using R software (www.r-project.org; packages stats and nlme (*17*,*18*)) (Technical Appendix Table 5).

Correlation of virus titration ($\log_{10}$[$TCID_{50}$/ml]) and RT-qPCR (Cq values) assay results of swab and blood samples collected from pigs, wild boar, goats and sheep experimentally i.n. infected or contact-infected with PPRV were analyzed using Spearman nonparametric correlation for the calculation of Spearman coefficients (rs) and *p* values (Technical Appendix Table 6). Therefore, results of oronasal, conjunctival and fecal swab samples and leukocytes were analyzed together, but separately by i) animal species, ii) animal trial, iii) detection method and iv) until versus after seroconversion. Spearman nonparametric correlation analysis was used since D'Agostino & Pearson normality test generally showed no normal distribution of the data (except for PCR results obtained from swab samples collected from goats (trial 3) and pigs (trial 1) after seroconversion). Correlation analysis (Technical Appendix Table 6) and visualization (Technical Appendix Figure 2) and D'Agostino & Pearson normality test were conducted with GraphPad Prism 7.02 software (GraphPad Software, Inc., CA, USA).

For all statistical analyses, *p*-values of $\alpha<0.05$ were considered statistically significant.

Suggested terminology and semantic of host status

A reservoir may be a maintenance or spillover host, independent from species, but depending on (multi-)host-pathogen-environment interactions (*19–21*). In maintenance hosts, disease persists without an external source of reinfection, while disease in spillover hosts will disappear if the source of infection is eliminated. However, although spillover hosts may be dead-end hosts, they may occasionally play an important epidemiologic role by spilling a pathogen back to a maintenance host or by spilling the pathogen forward to another spillover host (*19*,*20*,*22*).

In a multi-host context, the basic reproductive rate (R_0, the number of new or secondary cases of infection within a host population previously naïve to the pathogen of concern) can potentially be topped up by inter-species transmissions from other species that are maintenance or non-maintenance hosts (*20*). In contrast, if the number of individuals is low, extinction of the pathogen may easily occur even if R_0 of the pathogen in the new landscape is greater than R_0 (*21*). Furthermore, R_0 may change, e.g., in different environments or due to a change in the pathogen traits (*21*). Hence, the epidemiologic role of a species may not be definite but is rather dynamic considering persistence and transmission efficiency between host populations in the respective environment (*19*).

The drivers (risk factors) of pathogen persistence or emergence may include population density, social behavior, animal management (e.g., intra- and inter species interaction in the wild or in a flock of a farmer or a nomad, exploitation of natural resources and sharing of water wholes or feeding places, and defecation habits), encroachment of wildlife habitats at the wildlife/agricultural interface, animal breed and changes in the pathogen resistance or phenotype (e.g., driven by genetic changes or different environmental or host conditions) (*19*–*21*,*23*).

Another key factor may be that individual animals may act as “superspreader” as, for example, described for *Middle East respiratory syndrome coronavirus* (MERS-CoV) (*24*) and *West Nile virus* (WNV) (*25*) infections in humans. Stress, concurrent pathogen infections, physiologic demands, climate change and land use were described as possible factors in migratory animals influencing the possibility to act as superspreaders that may infect a considerable number of susceptible hosts (*26*). Hence, individual animals in a population of spillover hosts may act as important amplifying hosts that spill back or spill forward the pathogen to the maintenance or another spillover host, respectively.

According to Haydon et al. (*22*), control is ineffective if directed to reservoir components that are neither maintenance hosts nor transmitters of the pathogen to a defined target population (*22*), but this may change in different ecologic settings. Most efficient disease control efforts are aimed at maintenance hosts but may additionally require reduction or elimination of spillback (*19*,*20*).

For PPRV epidemiology in a multi-host system, we therefore propose to use the terminology of a dynamic multi-host-pathogen-environmental system (*21*) including spillover and maintenance hosts as suggested by Nugent (*20*) and Palmer et al. (*19*).

Results

Contact transmission

Of trial 1, two (P2 and P3) of the three experimental inoculated pigs were infected due to the experimental infection with PPRV, while one contact-control goat (G5) and very likely one (P1) of the three pigs were contact-infected about 1 week pi (estimated at 8 dpi) by P2 and/or P3. The second contact-control goat (G4) was found PPRV-infected about 1 week later (estimated at 16 dpi). G5 and P1 shared the food in the feeding trough with the other pigs, while G4 avoided

contact with the pigs and did not obviously feed together with the pigs, but from a separate feeding trough.

Contact transmission from the two experimentally PPRV-infected goats (G7 and G8) of trial 3 to the two contact-control pigs (P5 and P6) could be detected in both pigs at 6 dpi (Figure 1) - shortly after the first detection of infectious PPRV in goat swab samples. According to serologic and virological analyses, the two pigs were contact-infected a few days earlier (estimated at 4 dpi) than the P1 of trial 1 (estimated at 8 dpi).

During the wild boar and sheep trials, PPRV was not transmitted to any of the contact goats or pigs, although infectious PPRV was detected by virus isolation in some swab samples of secretions or excretions.

Clinical signs after PPRV-infection

The progression of clinical signs in the four different animal species are shown schematically in Figure 1 and in detail in Technical Appendix Figure 1.

Pigs and wild boar

The progression of clinical signs in the four different animal species are shown schematically in Figure 1 and in detail in Technical Appendix Figure 1. In trial 1, P1 was very likely refractory to PPRV-infection after experimental inoculation due to clinical, serologic and virological results and was therefore considered a contact control animal in this trial. Accordingly, the three pigs P1 (trial 1), P5 and P6 (trial 3) were infected with PPRV by contact during the transmission trials at ≈8 dpi and 4 dpi, respectively. To facilitate the comparison of clinical signs with the experimentally i.n. infected pigs (P2, P3) and wild boar (W1 to W4), the day of contact infection (dpci) is given for the three contact-infected pigs.

All three i.n. inoculated pigs of trial 1 showed a marked transient rise in body temperature >40°C (max. 41.0°C) at single days (P3 at 4 dpi, P2 at 8dpi) or for 4 consecutive days (P1 at 5 to 8 dpci), ruffling bristles (P3 at 8 dpi, P1 at 7 dpci), a reduced activity and food intake / slight emaciation (P3 at 7 dpi / 8dpi), swelling of the eye lids and mild to severe conjunctivitis (P2 at 8 to 12 dpi, P3 at 9 to 16 dpi, P1 at 7 to 15 dpci), as well as mucous to purulent ocular discharge in the first days after infection. Most severe conjunctivitis together with mucopurulent ocular discharge was seen in P2 and P3 at 11 dpi and in P1 at 10 dpci. One of the experimentally PPRV-infected pigs (P3) had diarrhea for a single day (at 8 dpi). A maximum

clinical score of 4, 3 or 6 was found at 8 dpi (P2 and 3) and 7dpci (P1). The pigs were reconvalescent after 13 dpi (P2), 17 dpi (P3) or 19dpci (P1). Hence, the clinical signs of the pig PPRV-infected by contact transmission (P1) were presented at a similar stage after PPRV-infection as previously observed in the experimentally PPRV-infected pigs (P2 and P3).

In the noninfected contact control pigs P5 and P6 of trial 2, no to mild clinical signs (clinical score of 0 to 2) possibly due to an increased rectal body temperature (up to 40.1°C) were observed at multiple days during the animal trial. From 6 to 8 dpci, the two pigs were mildly inactive (prolonged recumbency), mildly depressed and mildly inapparent. P5 additionally showed mucopurulent nasal discharge a single day (17 dpci).

In P5 and P6 of trial 3, a marked increase in rectal body temperature was found between 6 and 7 or 6 and 8 dpci, respectively (up to 40.3°C). At the same time both pigs showed a reduced appetite (6 to 8 dpci) and a reduced general condition (prolonged recumbency) (8 dpci). From 9 dpci, both pigs were reconvalescent.

In wild boars W1, W3 and W4 of trial 2, a marked increase in rectal body temperature was documented during the first weak after infection (40.2 to 40.9°C) and in W2 at 11 dpi (40.4°C) and 15 dpi (41.1°C). Shortly before experimental PPRV infection of the wild boars and after 9 dpi, an increase in body temperature >39.6°C was found at multiple days in three of the wild boars (W1, 2, 3). W4 and W1 showed pasty to watery diarrhea from 4 respectively 8 dpi until the end of the experiment. In addition, W4 had fresh blood in watery diarrhea at 7dpi. In W2 and W3, pasty to watery diarrhea was found at 7 and 14 dpi, respectively. W2 again showed diarrhea at 22 and 24 dpi and W3 at 17 dpi. All wild boar showed a mildly reduced general condition at 5 dpi, W3 additionally between 7 and 9 dpi and W2 at 25 dpi. W1 was mildly inappetent at 20 and 21 dpi, W2 at 24 dpi, and W3 at 15 dpi. W4 had mucopurulent nasal discharge at 8 and 9 dpi, P5 at 21 dpi. Peak clinical scores of 4 to 7 in the four wild boars were found at considerably different days (W1 at 21 dpi, W2 at 26 dpi, W3 at 15 dpi and W4 at 8 dpi).

Sheep

All sheep (S1, 3, 5, 7, 9) of trial no. 4 experimentally infected with PPRV showed a marked increase in rectal body temperature >40.0°C (max. 40.9°C; S9 at 7 and 8 dpi), in particular between 4 and 10 dpi. Mild to moderate clinical signs were found in 4 of the 5 sheep (peak clinical score of 5 between 4 and 10 dpi). In general, clinical signs in these 4 sheep

included serous to mucopurulent nasal discharge and pasty feces. One of the 5 sheep (S9) was more severely affected (peak clinical score of 14 at 10 dpi). S9 additionally showed respiratory distress, reddened/congested oral mucosa, open lesions in oral cavity, edematous lips, conjunctivitis, reduced general condition (prolonged recumbency) between 8 to 12 dpi. However, mild but similar clinical signs were also recorded in the contact control sheep (S2, 4, 6, 8, 10) on 2 to 4 single days (between 2 and 17 dpi), while S10 (the contact control sheep of S9) showed up to moderate clinical signs (max. clinical score of 5 at 10 dpi). Hence, except for S9 that showed temporary oral lesions, it remains unclear whether the clinical signs in the other i.n. infected sheep were due to PPRV infection or may have been aggravated by previous infection or vice versa.

Virological results after PPRV-infection

Pigs and wild boar

In the pigs, PPRV-RNA was generally detected in whole-blood, serum, leukocytes, urine, oronasal, conjunctival and fecal swabs for up to 4 weeks after experimental or contact infection (Technical Appendix Figure 1.1). In swab samples of pigs P1 to P3, PPRV-RNA copy numbers peaked between 5 and 8 dpi or dpci in oronasal (max. in P3 at 6 dpi, 4.3×10^6 copies/ml, Cq 25.99), conjunctival (max. in P3 at 7 dpi, 5.9×10^6 copies/ml, Cq 25.51) and fecal (max. in P3 at 7 dpi, 1.5×10^7 copies/ml, Cq 24.11) swabs. The median PPRV-RNA copy numbers in swabs from all three pigs between 2 and 30 dpi were 1.4×10^4, 3.9×10^3 and 1.6×10^4 copies/ml in oronasal, conjunctival and fecal swabs, respectively. Similarly, in whole-blood, serum and leukocytes (highest viral loads with Cq 29.1, 29.5 respectively 35.6), PPRV-RNA was detected during the first 10 days after experimental or contact infection, approximately at the time of seroconversion, as measured with cELISA. In urine, PPRV-RNA was detected at two or three of the 4 days of urine collection (except at post-mortem examination) between 8 and 10 or 14 dpi in P2 and P3, respectively. The highest viral PPRV-RNA loads were found in urine pellets at 8 dpi (Cq 32 to 33). In the urine pellet, viral loads were ≈0.5 log steps higher than in whole urine or urine supernatant.

PPRV was isolated with VDS and/or CHS-20 cells from conjunctival (peak of $10^{3.5}$ $TCID_{50}$/ml at 7 dpi) and fecal (peak of $10^{3.5}$ $TCID_{50}$/ml at 6 dpi) swabs from one of the experimentally infected pigs (P3) and from leukocytes from two of the pigs (P1 and P3) between

4 and 7 dpi, but not from oronasal swabs and urine samples (however, urine was collected around seroconversion). From leukocytes, PPRV was isolated at 4 and 6 dpi (P3) with a peak titer of 2.3 $log_{10}(TCID_{50}/10^6$ cells) at 6 dpi or at 14 dpi/6 dpci (P1) with a peak titer of 1.1 $log_{10}(TCID_{50}/10^6$ cells). After seroconversion at 8 dpi, no PPRV was isolated from any of the PPRV-RNA positive samples from the pigs.

In wild boar of trial no. 2 (Technical Appendix Figure 1.2), oronasal (2 to 14 dpi), conjunctival (5 to 15 dpi) and fecal (4 to 24 dpi) swabs were PPRV-RNA positive for up to 3 weeks after experimental infection with peak viral RNA loads between 6 and 8 dpi (W1, 3, 4) or 10 dpi (W2). Peak viral RNA loads were considerably higher in fecal (Cq 25.09 to 26.06) compared to oronasal (Cq 28.35 to 33.16) and conjunctival (Cq 30.04 to 37.72) swab samples. In whole blood samples, PPRV-RNA was only detected until seroconversion (up to 10 dpi). Similarly, PPRV could only be isolated from a few fecal samples with moderately high viral loads from W2 (at 5 and 6 dpi, $10^{2.0}$ and $10^{3.5}$ $TCID_{50}$/ml, Cq 29.39 and 26.50) and W4 (8,9 and 10 dpi, $10^{2.0}$ to $10^{2.5}$ $TCID_{50}$/ml, Cq 30.12 to 26.06) and from purified WBCs at 4 dpi (W1, 3, 4), 7 dpi (W3) or 10 dpi (W2), (1.0 to 2.2 $log_{10}(TCID_{50}/10^6$ cells), Cq 31.05 to 37.22) until seroconversion.

In the four in contact-animals P5, P6, G7 and G8 of the wild boar in trial 2, several intermittently PCR-positive results were obtained from oronasal (3 to 17 dpi), conjunctival (7 to 9 dpi) and fecal (7 to 8 dpi) swabs during the peak of viral excretion in wild boar. An explanation is the contamination of the stable with PPRV-(RNA) due to the high viral loads shed during that time by the wild boar.

In the contact-infected pigs P5 and P6 of trial 3, PPRV-RNA was detected in almost all swab samples from 6 dpi until euthanasia at 21 dpi/17 dpci. Viral RNA loads peaked at 10 dpi/8 dpci in oronasal (Cq 28.86 to 29.18), conjunctival (Cq 30.52 to 31.72) and fecal (Cq 30.63 to 31.22) swabs. In whole-blood, PPRV-RNA was detected between 8 and 12 dpi/4 and 8 dpci (up to Cq 34.32 at 10 dpi), and in serum only at the peak of RNAemia in P6 at 10 dpi. No infectious PPRV could be isolated from any of the tested (8 to 14 dpi) PCR-positive swab samples of the contact-infected pigs P5 and P6 of trial 3.

Sheep

In sheep, PPRV-RNA was generally detectable from 2 or 4 dpi until euthanasia, but the period of time varied considerably between individual animals (Technical Appendix Figure 1.4). For S1, only a few oronasal swab samples (4 to 8 dpi), one fecal swab (12 dpi) and one whole blood sample (8 dpi) were found PCR-positive (Cq >36.63), while the other 4 sheep showed PCR-positive swab samples (S3, 5, 7, 9) and whole blood samples (S3, 7, 9) until euthanasia at 21 dpi. In sheep S3, 5, 7 and 9, viral RNA loads peaked between 6 and 10 dpi in oronasal swabs (Cq 22.37 to 24.99) and at 8 or 10 dpi in conjunctival (Cq 24.09 to 27.86) and in fecal (24.09 to 27.86) swabs. In serum from 3 of 5 sheep (S3,5,7), PPRV-RNA was detected at 6 and/or 8 dpi (Cq 38), while whole blood samples were PCR-positive from 4 or 6 dpi until euthanasia at 21 dpi for 1 to 17 consecutive days with peak viral loads between Cq 30.14 and 32.30.

PPRV was isolated from sheep swab samples at single or multiple consecutive days between 4 and 10 dpi - until seroconversion at 8 dpi or shortly after seroconversion (10 dpi, S9). Highest PPRV loads varied between $10^{2.5}$, $10^{3.0}$ and $10^{4.0}$ TCID50/ml in fecal, conjunctival and oronasal swabs, respectively, and were isolated from samples with Cq values between 22.37 and 37.14, 23.67 and 32.90 or 24.09 and 32.39 in oronasal, conjunctival and fecal swabs, respectively. From S1, only a single sample was found positive for PPRV (oronasal swab at 4 dpi, $10^{2.0}$ TCID50/ml, Cq 37.14). No virus isolation was conducted from sheep leukocytes.

References

1. Hoffmann B, Wiesner H, Maltzan J, Mustefa R, Eschbaumer M, Arif FA, et al. Fatalities in wild goats in Kurdistan associated with Peste des Petits Ruminants virus. Transbound Emerg Dis. 2012;59:173–6. PubMed http://dx.doi.org/10.1111/j.1865-1682.2011.01270.x

2. Wernike K, Eschbaumer M, Breithaupt A, Maltzan J, Wiesner H, Beer M, et al. Experimental infection of sheep and goats with a recent isolate of peste des petits ruminants virus from Kurdistan. Vet Microbiol. 2014;172:140–5. PubMed http://dx.doi.org/10.1016/j.vetmic.2014.05.010

3. Adombi CM, Lelenta M, Lamien CE, Shamaki D, Koffi YM, Traoré A, et al. Monkey CV1 cell line expressing the sheep-goat SLAM protein: a highly sensitive cell line for the isolation of peste des petits ruminants virus from pathological specimens. J Virol Methods. 2011;173:306–13. PubMed http://dx.doi.org/10.1016/j.jviromet.2011.02.024

4. von Messling V, Springfeld C, Devaux P, Cattaneo R. A ferret model of canine distemper virus virulence and immunosuppression. J Virol. 2003;77:12579–91. PubMed http://dx.doi.org/10.1128/JVI.77.23.12579-12591.2003

5. Pope RA, Parida S, Bailey D, Brownlie J, Barrett T, Banyard AC. Early events following experimental infection with Peste-Des-Petits ruminants virus suggest immune cell targeting. PLoS One. 2013;8:e55830. PubMed http://dx.doi.org/10.1371/journal.pone.0055830

6. Heinritzi K. Besonderheiten bei der speziellen Untersuchung des Schweines. In: Heinritzi K, Gindele HR, Reiner G, Schnurrbusch U, editors. Schweinekrankheiten. Pustet, Regensburg: Eugen Ulmer KG; 2006. p. 23–7.

7. Mittelholzer C I, Moser C II, Tratschin JD, Hofmann MA. Analysis of classical swine fever virus replication kinetics allows differentiation of highly virulent from avirulent strains. Vet Microbiol. 2000;74:293–308. PubMed http://dx.doi.org/10.1016/S0378-1135(00)00195-4

8. Plonait H. Fieberhafte Allgemeinerkrankungen. In: Waldmann KH, Wendt M, editors. Lehrbuch der Schweinekrankheiten. 3 ed. Berlin: Parey Buchverlag im Blackwell Wissenschafts-Verlag GmbH; 2001. p. 93–104.

9. Mayr A, Bachmann PA, Bibrack B, Wittmann G. Quantitative Bestimmung der Virusinfektiosität (Virustitration). In: Mayr A, Bachmann PA, Bibrack B, Wittmann G, editors. Virologische Arbeitsmethoden Band I. Jena: VEB Gustav Fischer Verlag; 1974. p. 35–9.

10. Dagur PK, McCoy JP, Jr. Collection, Storage, and Preparation of Human Blood Cells. Curr Protoc Cytom. 2015;73:5 1 –16. http://dx.doi.org/10.1002/0471142956.cy0501s73

11. Hoffmann B, Depner K, Schirrmeier H, Beer M. A universal heterologous internal control system for duplex real-time RT-PCR assays used in a detection system for pestiviruses. J Virol Methods. 2006;136:200–9. PubMed http://dx.doi.org/10.1016/j.jviromet.2006.05.020

12. Bréard E, Schulz C, Sailleau C, Bernelin-Cottet C, Viarouge C, Vitour D, et al. Bluetongue virus serotype 27: Experimental infection of goats, sheep and cattle with three BTV-27 variants reveal atypical characteristics and possible direct contact transmission BTV-27 between goats. Transbound Emerg Dis. In press.

13. Schulz C, Sailleau C, Bréard E, Flannery J, Viarouge C, Zientara S, et al. Experimental infection of sheep, goats and cattle with a bluetongue virus serotype 4 field strain from Bulgaria, 2014. Transbound Emerg Dis. 2018;65:e243–50. PubMed http://dx.doi.org/10.1111/tbed.12746

14. Batten CA, Banyard AC, King DP, Henstock MR, Edwards L, Sanders A, et al. A real time RT-PCR assay for the specific detection of Peste des petits ruminants virus. J Virol Methods. 2011;171:401–4. PubMed http://dx.doi.org/10.1016/j.jviromet.2010.11.022

15. Bao J, Li L, Wang Z, Barrett T, Suo L, Zhao W, et al. Development of one-step real-time RT-PCR assay for detection and quantitation of peste des petits ruminants virus. J Virol Methods. 2008;148:232–6. PubMed http://dx.doi.org/10.1016/j.jviromet.2007.12.003

16. Polci A, Cosseddu GM, Ancora M, Pinoni C, El Harrak M, Sebhatu TT, et al. Development and Preliminary Evaluation of a New Real-Time RT-PCR Assay For Detection of Peste des petits Ruminants Virus Genome. Transbound Emerg Dis. 2013. PubMed

17. R Core Team. R: A Language and Environment for Statistical Computing. The R Foundation for Statistical Computing. Version 3.2.0. Vienna, Austria. 2015 [cited 2018Oct 12]. http://www.R-project.org/

18. Pinheiro J, Bates D. DebRoy S, Sarkar D, R Core Team. nlme: Linear and Nonlinear Mixed Effects Models. R package version 3.1–131. 2017 [cited 2018 Oct 12]. https://CRAN.R-project.org/package=nlme

19. Palmer MV, Thacker TC, Waters WR, Gortazar C, Corner LA. Mycobacterium bovis: A Model Pathogen at the Interface of Livestock, Wildlife, and Humans. Vet Med Int. 2012;2012:236205. http://dx.doi.org/10.1155/2012/236205

20. Nugent G. Maintenance, spillover and spillback transmission of bovine tuberculosis in multi-host wildlife complexes: a New Zealand case study. Vet Microbiol. 2011;151:34–42. 10.1016/j.vetmic.2011.02.023 PubMed http://dx.doi.org/10.1016/j.vetmic.2011.02.023

21. Engering A, Hogerwerf L, Slingenbergh J. Pathogen-host-environment interplay and disease emergence. Emerg Microbes Infect. 2013;2:e5. PubMed http://dx.doi.org/10.1038/emi.2013.5

22. Haydon DT, Cleaveland S, Taylor LH, Laurenson MK. Identifying reservoirs of infection: a conceptual and practical challenge. Emerg Infect Dis. 2002;8:1468–73. PubMed http://dx.doi.org/10.3201/eid0812.010317

23. EFSA. European Food Safety Authority (EFSA). Scientific Opinion on peste des petits ruminants. EFSA Panel on Animal Health and Welfare (AHAW). EFSA J. 2015;13:3985.

24. Am H, Alqahtani AS, Alaklobi F, Altayeb J, Memish ZA. Healthcare worker exposure to Middle East respiratory syndrome coronavirus (MERS-CoV): Revision of screening strategies urgently

needed. International journal of infectious diseases: IJID: official publication of the International Society for Infectious Diseases. 2018;71:113–6. http://dx.doi.org/10.1016/j.ijid.2018.04.001

25. Gervasi SS, Burgan SC, Hofmeister E, Unnasch TR, Martin LB. Stress hormones predict a host superspreader phenotype in the West Nile virus system. Proc Biol Sci. 2017;284:20171090. 10.1098/rspb.2017.1090 PubMed http://dx.doi.org/10.1098/rspb.2017.1090

26. Fritzsche McKay A, Hoye BJ. Are Migratory Animals Superspreaders of Infection? Integr Comp Biol. 2016;56:260–7. 10.1093/icb/icw054 PubMed http://dx.doi.org/10.1093/icb/icw054

Technical Appendix Table 1. Scientific names, breeds, sex and age of the study animals of the PPRV transmission trials. Further details on the study design are given in the Table in the main article.

		Intranasally inoculated animals			Contact control animals		
**Trial no.**	**Trial ID**	**Species* and ID**	**Sex**	**Age (months)**	**Species* and ID**	**Sex**	**Age (months)**
1	P-GP	P1-P3†	3M	2	G4, G5, P1†	2M	3
2	W-GP	W1-W4	3F, 1M	3	G7, G8	2M	3
					P5, P6	2F	3
3	G-P	G7, G8	2M	4–5	P5, P6	2F	4
4	S-S	S1, S3, S5, S7, S9	5M	4	S2, S4, S6, S8, S10	5M	4

*scientific names and breeds: pig (P) (*Sus scrofa domesticus*; breed: hybrid of German Landrace and Large White; *german*: ‘Deutsche Landrasse’ respectively ‘Deutsches Edelschwein’), wild boar (W) (*Sus scrofa*), goat (G) (*Capra aegagrus hircus*; breed: White German goat; *german*: ‘Weiße Deutsche Edelziege’), sheep (S) (*Ovis aries*; breed: Merinoland sheep; *german*: Merinolandschaf); M, male; F, female; †1 (P1) of 3 pigs was probably not infected by experimental intranasal PPRV-inoculation but by contact-infection

Technical Appendix Table 2. Clinical Score (CS) sheet for the evaluation of clinical signs in PPRV-infected pigs and wild boar. Table adapted for suids according to Heinritzi (6), Mittelholzer et al. (7), Plonait (8) using the clinical score sheet for PPRV-infected small ruminants proposed by Pope et al. (5).

Clinical Score	General signs	Rectal body temperature (°C)	Ocular/nasal discharge	Facial mucosal lesions	Faeces	Respiratory symptoms
**0**	Normal (alert, curious, promptly stands up)	<39,5	None	None	Normal (*formed*)	Normal respiration rate (*10–25/min), scarcely visible thoracic movement*
**1**	Mildly inactive (somewhat tired)	>39,5 but ≤40	Watery ocular discharge	Congested oronasal mucosa and buccal papillae	Mild diarrhea	Mild tachypnoea
**2**	Mildly inactive and depressed, mild inappetance	>40 but ≤41	Watery to mucoid oculonasal discharge: reddened eyes and mild conjunctivitis	Pin-prick lesions within buccal cavity, with some becoming more extensive	Runny	Tachypnoea/ mild cough, *marked thoracic movement*
**3**	inactive, apathetic, restless and anorexic	>41 or >39.5 for >5 d	Mucopurulent nasal discharge and/or severe conjunctivitis with mucopurulent ocular discharge	Clear erosive lesions on oronasal mucosae; severely congested/oedematous buccal papillae	Frank diarrhea	Tachypnoea and dyspnoea/ coughing
**4**	Severe depression, unable to stand, extreme lethargy, dehydration	>41 or >39.5 for >5 d followed by rapid fall of temperature (*<38.5*)	Mucopurulent nasal discharge and severe conjunctivitis with profuse mucopurulent ocular discharge	Severe erosive/ulcerative lesions throughout buccal cavity, nasal mucosa and nares; oedematous lips and erosions on vulval labia	Muco-haemorrhagic diarrhea	Marked tachypnoea / dyspnoea/ cough

End point definition: When animal reaches a score of 20 they need to be killed on ethical grounds. The decision to euthanize would additionally be based on the following criteria: 1) A score of 4 is achieved in „General Signs“; 2) A score of 3 is achieved in „General Signs“ for 2 complete, consecutive days and score of 10 or greater is achieved in other categories; 3) A score of 2 is achieved in „General Signs“ for 2 complete, consecutive days and score of 15 or greater is achieved in other categories. CS criteria in italic were modified from the CS sheet published by Pope et al. (5) according to Heinritzi (6), Mittelholzer et al. (7), Plonait (8).

Technical Appendix Table 3. Selection of real-time quantitative reverse transcription-PCR (RT-qPCR) and immunohistochemical (IHC) results of tissue samples collected during PPRV-transmission trials from pigs (P), wild boar (W), goats (G) and sheep (S). The animals were experimentally (pi) or contact (pci) infected with PPRV lineage IV strain Kurdistan/2011 (details about the study designs of the transmission trials are given in the table in the main article). RT-qPCR results were determined with the PPRV PCR assay of Batten et al. (14) and IHC results using Mab anti-PPRV-Np (purified monoclonal mouse antibody against PPRV nucleoprotein, ID.vet). Tissue most suitable for PPRV diagnosis in all species are highlighted in bold. The negative control goat and sheep were both PPR-negative with PCR and IHC (data not shown)*

Technical Appendix Table 3, part A

		**animal ID**	**P1**	**P2**	**P3**	**P5**	**P6**	**W1**	**W2**	**W3**	**W4**
		**animal trial ID (no.)**	**P-GP (1)**	**P-GP (1)**	**P-GP (1)**	**G-P (3)**	**G-P (3)**	**W-GP (2)**	**W-GP (2)**	**W-GP (2)**	**W-GP (2)**
		**infection status**	**inci**	**in**	**in**	**cci**	**cci**	**in**	**in**	**in**	**in**
		**dpi (dpci)**	**30 (22)**	**16**	**30**	**21 (17)**	**21 (17)**	**28**	**28**	**28**	**28**
**organ location**	**organ ID**	**tissue (Cq/IHC)**									
head	1	third eye lid*	–/–	–/–	–/–	–/–	-/nd	–/–	–/–	-/+	+/–
	2	lacrimal gland	–/–	–/–	–/–	-/++	-/+	-/nd	–/–	-/+	-/+
	6	tongue (apex)	-/nd	nd/nd	nd/nd	-/nd	-/nd	-/nd	-/nd	-/nd	+/nd
	7	**tonsil***	+/+	+/++	+/–	++/+++	++/+	++/–	++/–	++/++	++/+
	8	**retropharyngeal ln.**	–/–	–/–	+/–	++/++	++/–	++/–	–/–	+/+	-/+
	9	**mandibular ln.**	nd/nd	nd/nd	nd/nd	+/++	+/+	++/–	+/–	+/+	++/+
	10	**parotideal ln.**	nd/nd	nd/nd	nd/nd	++/++	++/–	+/–	+/–	+/+	+/+
cervical†	12	esophagus	–/–	–/–	–/–	–/–	–/–	–/–	–/–	–/–	+/–
thoracal	13	lung	–/–	–/–	–/–	–/–	–/–	–/–	–/–	-/+	–/–
	14	**bronchial ln.**	–/–	–/–	+/–	+/–	++/–	++/–	–/–	–/–	+/+
	15	**mediastinal ln.**	–/–	–/–	–/–	+/–	++/–	++/–	+/–	+/+	–/–
	17	thymus	nd/nd	nd/nd	nd/nd	nd/nd	nd/nd	-/nd	-/nd	+/nd	-/nd
abdominal	19	**jejunal PP**	+/–	+/–	–/–	++/+++	++/–	–/–	++/–	+/++	–/–
	20	**ileal PP**	–/–	–/–	–/–	++/+++	++/–	+/–	++/–	+/+	++/++
	22	Colon*	–/–	+/–	–/–	+/+	-/+	–/–	–/–	-/+	-/++
	23	**mesenterial ln.**	–/–	–/–	–/–	++/++	++/+	++/–	++/–	+/+	+/–
	24	Rectum*	–/–	+/–	–/–	-/+	++/+	–/–	–/–	-/+	–/–
	27	spleen	–/–	-/nd	–/–	+/+	+/–	+/nd	+/–	+/+	+/–
cerebral	30	choroid plexus	-/nd	-/nd	-/nd	-/nd	+/nd	-/nd	-/nd	-/nd	+/nd
		olfactory nerve	-/nd	-/nd	-/nd	-/nd	-/nd	-/nd	-/nd	-/nd	-/nd
		optic nerve	-/nd	-/nd	-/nd	-/nd	-/nd	-/nd	-/nd	-/nd	-/nd
		optic chiasm	-/nd	nd/nd	-/nd	-/nd	-/nd	-/nd	-/nd	-/nd	-/nd
		pons	-/nd	-/nd	-/nd	-/nd	-/nd	-/nd	-/nd	-/nd	-/nd
		white cerebellum	-/nd	-/nd	-/nd	nd/nd	nd/nd	nd/nd	nd/nd	nd/nd	nd/nd
		white cerebrum	nd/nd	nd/nd	nd/nd	-/nd	-/nd	-/nd	-/nd	-/nd	-/nd
		fourth ventricle (roof)	-/nd	-/nd	-/nd	-/nd	-/nd	-/nd	-/nd	-/nd	-/nd
		spinal cord (thoracical)	-/nd	-/nd	-/nd	-/nd	-/nd	-/nd	-/nd	-/nd	-/nd
		medulla oblongata	-/nd	-/nd	-/nd	-/nd	-/nd	-/nd	-/nd	-/nd	-/nd
		trigeminal ganglion	-/nd	nd/nd	-/nd	-/nd	-/nd	-/nd	-/nd	-/nd	-/nd
genito-urinary tract	31	testical	nd/nd	nd/nd	nd/nd	nd/nd	nd/nd	nd/nd	nd/nd	nd/nd	-/nd
		epididymis	nd/nd	nd/nd	nd/nd	nd/nd	nd/nd	nd/nd	nd/nd	nd/nd	-/nd
		urine	-/nd	-§/nd	-§/nd	nd/nd	-/nd	-/nd	nd/nd	-/nd	-/nd
muscular	32	musculus (m.) gracilis	nd/nd	nd/nd	nd/nd	nd/nd	nd/nd	nd/nd	nd/nd	nd/nd	nd/nd
		m. longissimus dorsi	nd/nd	nd/nd	nd/nd	nd/nd	nd/nd	nd/nd	nd/nd	nd/nd	nd/nd

Technical Appendix Table 3, part B

organ location	organ ID	animal ID<br>animal trial no. (ID)<br>infection status<br>dpi (dpci)<br>tissue (Cq/IHC)	G4<br>P-GP (1)<br>cci<br>30 (15)	G5<br>P-GP (1)<br>cci<br>19 (11)	G7<br>G-P (3)<br>in<br>12	G8<br>G-P (3)<br>in<br>12	S1<br>S-S (4)<br>in<br>21	S3<br>S-S (4)<br>in<br>21	S5<br>S-S (4)<br>in<br>21	S7<br>S-S (4)<br>in<br>21	S9<br>S-S (4)<br>in<br>21
head	1	third eye lid*	+/–	+++/+++	++++/++	+++/–	+/–	++/nd	++/–	++/–	++/–
	2	lacrimal gland	++/–	+++/–	+++/–	++++/++	–/–	+/nd	-/nd	+/–	+/–
	6	tongue (apex)	nd/nd	++++/nd	+++/nd	+++/nd	nd/nd	-/nd	nd/nd	+/nd	+/nd
	7	**tonsil***	++/nd	+++/nd	++++/nd	+++/++	++/+	++/nd	+/+	++/–	++/–
	8	**retropharyngeal ln.**	++/–	+++/+++	+++/++	+++/+++	–/–	++/nd	++/+	++/–	++/–
	9	**mandibular ln.**	nd/nd	nd/nd	+++/–	+++/–	-/nd	++/–nd	++/–	+++/–	++/–
	10	**parotideal ln.**	nd/nd	nd/nd	+++/++	++/+++	++/–	++/nd	+/–	++/–	++/–
cervical†	12	esophagus	–/–	+++/–	++/–	++/–	nd/nd	-/nd	nd/-	+/–	–/–
thoracal	13	lung	+++/–	+++/+++	+++/+++	+++/+++	-/+	-/nd	–/–	+/–	+/+
	14	**bronchial ln.**	++/–	+++/+++	+++/++	+++/–	–/–	++/nd	++/–	++/–	++/+
	15	**mediastinal ln.**	++/–	+++/+++	+++/+++	+++/–	+/–	++/nd	++/+	++/–	++/–
	17	thymus	nd/nd	nd/nd	nd/nd	nd/nd	nd/nd	nd/nd	nd/nd	nd/nd	nd/nd
abdominal	19	**jejunal PP**	+/–	+++/++++	++++/+++	++++/++++	–/–	-/nd	++/–	++/–	–/–
	20	**ileal PP**	++/–	++++/++++	++++/++++	++++/++++	+/–	++/nd	–/–	++/–	++/–
	22	Colon*	++/–	+++/++	++++/nd	++++/nd	–/–	+/nd	–/–	–/–	–/–
	23	**mesenterial ln.**	+/–	+++/++++	+++/+++	+++/++	–/–	++/nd	++/–	++/–	++/–
	24	Rectum*	++/–	+++/++	++++/++++	++++/++++	nd/nd	-/nd	nd/nd	+/nd	++/–
	27	spleen	++/–	+++/++	+++/–	++/–	–/–	++/nd	++/–	++/–	++/–
cerebral	30	choroid plexus	-/nd	++/nd	+++/nd	+++/nd	nd/nd	-/nd	nd/nd	-/nd	-/nd
		olfactory nerve	-/nd	++/nd	++/nd	++/nd	nd/nd	+/nd	nd/nd	-/nd	+/nd
		optic nerve	-/nd	++/nd	++/nd	+/nd	nd/nd	+/nd	nd/nd	-/nd	-/nd
		optic chiasm	-/nd	+++/nd	++/nd	++/nd	nd/nd	/nd	nd/nd	-/nd	+/nd
		pons	-/nd	+/nd	++/nd	++/nd	nd/nd	+/nd	nd/nd	-/nd	+/nd
		white cerebellum	-/nd	+/nd	nd/nd	nd/nd	nd/nd	nd/nd	nd/nd	nd/nd	nd/nd
		white cerebrum	-/nd	++/nd	++/nd	++/nd	nd/nd	-/nd	nd/nd	-/nd	-/nd
		fourth ventricle (roof)	-/nd	++/nd	++/nd	++/nd	nd/nd	-/nd	nd/nd	-/nd	+/nd
		spinal cord (thoracical)	+/nd	++/nd	-/nd	+/nd	nd/nd	-/nd	nd/nd	-/nd	-/nd
		medulla oblongata	-/nd	++/nd	++/nd	+++/nd	nd/nd	+/nd	nd/nd	-/nd	-/nd
		trigeminal ganglion	-/nd	++/nd	++/nd	++/nd	nd/nd	-/nd	nd/nd	-/nd	-/nd
genito-urinary tract	31	testical	nd/nd	nd/nd	-/nd	++/nd	nd/nd	-/nd	nd/nd	-/nd	-/nd
		epididymis	nd/nd	nd/nd	++/nd	++/nd	nd/nd	-/nd	nd/nd	-/nd	-/nd
		urine	+/nd	+++/nd	+++/nd	++++/nd	nd/nd	-/nd	nd/nd	++/nd	++/nd
muscular	32	musculus (m.) gracilis	-/nd	++/nd	nd/nd	nd/nd	nd/nd	-/nd	nd/nd	-/nd	-/nd
		m. longissimus dorsi	-/nd	++/nd	nd/nd	nd/nd	nd/nd	-/nd	nd/nd	-/nd	-/nd

*cci, infected by contact infection; Cq, quantitative cycle value; dpi, days after infection; dpci, estimated day after contact infection; P, pig; W, wild boar; G, goat; S, sheep; IHC, immunohistochemically detected PPR antigen; in, intranasally infected; inci, intranasally inoculated but infected by contact infection; ln., lymph node; PP, Peyer's patches; *for samples stained by IHC, results of lymph reticular system and parenchymal tissue (the higher positive results is presented) are summarized, individual results are shown iAppendix Table 5; †trachea were all negative by PCR and IHC except for all 4 goats (PCR: G4,5,7,8; PCR: G7,8); ++++, Cq 18–24.999, high PPRV-RNA load/IHC severe; +++, Cq 25–29.999, moderate PPRV-RNA load/IHC moderate; ++, Cq 30–34.999, low PPRV-RNA load/IHC mild; +, Cq 35–41, weak PPRV-RNA load/IHC weak; -, no Cq/IHC negative; nd, not done; §urine samples positive from P2 and P3 at 8 and 10 dpi (Cq + to ++).

Technical Appendix Table 4. Results of the comparison of different methods for virological PPRV diagnosis in animals of various *Artiodactyla* species. Therefore, different sample matrices (swab, tissue, blood) were analyzed from pigs (P), wild boar (W), goats (G) and sheep (S) experimentally infected or contact infected with the PPRV lineage 4 strain Kurdistan/2011 using two SLAM-expressing cell lines (VDS and/or CHS-20) for virus isolation, three PCR assays for real-time quantitative reverse transcription PCR (RT-qPCR), antigen ELISA (Ag-ELISA) and lateral flow device (LFD)

Serial no.	Animal trial ID (trial no.)	Infection status	Animal species	Sample material	Type	Animal ID	dpi (dpci)	SLAM-cells (max. $TCID_{50}$/ml [cell line])	Vero.dog.SLAM .tag ($TCID_{50}$/ml)	CHS-20 ($TCID_{50}$/ml)	Detection of PPRV-Np by RT-qPCR (Cq value)			Ag-ELISA (OD NC %)	LFD (pos/ neg)
											Bao et al. 2008	Polci et al. 2013	Batten et al. 2011		
1	goat TV 2-14	in	goat	Conjunctival	swab	G8	7	10^4.5 [CHS]*	10^4.0*	10^4.5*	23.54	23.65	21.71	108	neg
2	goat TV 2-14	in	goat	Conjunctival	swab	G10	7	10^4.5 [CHS]*	10^3.0*	10^4.5*	24.21	24.37	22.57	66	neg
3	goat TV 2-14	in	goat	Fecal	swab	G4	7	10^4.5 [CHS]*	10^3.5*	10^4.5*	23.39	22.13	20.09	107	neg
4	goat TV 2-14	in	goat	Fecal	swab	G8	7	10^3.5 [VDS]*	10^3.5*	neg*	26.17	25.03	22.96	69	neg
5	goat TV 2-14	in	goat	Fecal	swab	G9	7	10^2.0 [CHS]*	neg*	10^2.0*	29.76	29.66	27.46	-2	neg
6	goat TV 3-15	in	goat	Serum	serum	G1	6	*ND*	*ND*	*ND*	33.24	33.29	30.81	43	lgpos
7	goat TV 3-15	in	goat	EDTA-blood	blood	G1	8	*ND*	*ND*	*ND*	26.41	29.00	22.95	29	neg
8	goat TV 3-15	in	goat	Oronasal	swab	G1	8	10^5.0 [CHS]*	10^3.5*	10^5.0*	20.05	21.20	19.45	108	pos
9	goat TV 3-15	in	goat	Conjunctival	swab	G1	8	10^4.5 [CHS]*	10^3.5*	10^4.5*	19.80	21.09	19.41	108	pos
10	goat TV 3-15	in	goat	EDTA-blood	blood	G1	9	*ND*	*ND*	*ND*	27.21	30.12	28.58	19	neg
11	goat TV 3-15	in	goat	Serum	serum	G1	9	*ND*	*ND*	*ND*	33.49	34.35	35.90	28	lgpos
12	goat TV 3-15	in	goat	3. eye lid	tissue	G1	9	10^4.0 [CHS]*	neg*	10^4.0*	22.64	23.59	22.70	108	ND
13	goat TV 3-15	in	goat	Lacrimal gland	tissue	G1	9	neg*	neg*	neg*	20.76	22.54	20.74	92	ND
14	goat TV 3-15	in	goat	Conche	tissue	G1	9	10^4.0 [CHS]*	10^3.5*	10^4.0*	20.22	21.36	20.12	104	ND
15	goat TV 3-15	in	goat	Colon	tissue	G1	9	10^5.0 [CHS]*	10^4.5*	10^5.0*	18.45	19.16	17.88	113	ND
16	goat TV 3-15	in	goat	Bile fluid	tissue	G1	9	neg*	neg*	neg*	20.47	18.97	17.93	112	ND
17	P-GP (trial 1)	cci	goat	Oronasal	swab	G5	16 (8)	10^5.5 [VDS]	10^5.5	*ND*	24.72	22.42	21.69	110	pos
18	P-GP (trial 1)	cci	goat	leukocytes	wbc	G5	16 (8)	10^4.5 [VDS]	neg*	neg*	32.51	30.04	29.01	9	neg
19	P-GP (trial 1)	cci	goat	Oronasal	swab	G5	19 (11)	10^6.0 [VDS]	10^6.0	*ND*	22.13	20.88	19.96	110	pos
20	P-GP (trial 1)	cci	goat	Conjunctival	swab	G5	19 (11)	10^4.5 [VDS]	10^4.5	*ND*	23.03	21.42	20.64	109	lgpos

Serial no.	Animal trial ID (trial no.)	Infection status	Animal species	Sample material	Type	Animal ID	dpi (dpci)	SLAM-cells (max. TCID$_{50}$/ml [cell line])	Vero.dog.SLAM .tag (TCID$_{50}$/ml)	CHS-20 (TCID$_{50}$/ml)	Detection of PPRV-Np by RT-qPCR (Cq value)			Ag-ELISA (OD NC %)	LFD (pos/ neg)
											Bao et al. 2008	Polci et al. 2013	Batten et al. 2011		
21	P-GP (trial 1)	cci	goat	Urine	urine	G5	19 (11)	10^2.5 [VDS]	10^2.5	*ND*	29.11	29.59	27.53	72	neg
22	P-GP (trial 1)	cci	goat	Oronasal	swab	G4	24 (8)	10^2.5 [VDS]	10^2.5	*ND*	24.90	25.75	24.08	118	neg
23	P-GP (trial 1)	cci	goat	Fecal	swab	G4	24 (8)	10^5.0 [VDS]	10^5.0	*ND*	25.73	25.38	23.78	118	pos
24	P-GP (trial 1)	cci	goat	leukocytes	wbc	G4	24 (8)	10^4.0 [VDS]	neg*	neg*	32.31	32.38	30.63	2	neg
25	P-GP (trial 1)	in	pig	leukocytes	wbc	P3	4	10^3.5 [VDS]	10^2.5*	10^2.5*	30.33	30.64	29.18	11	neg
26	P-GP (trial 1)	in	pig	Conjunctival	swab	P3	5	10^3.0 [VDS]	neg*	10^2.0*	30.54	30.85	28.70	26	neg
27	P-GP (trial 1)	in	pig	Fecal	swab	P3	5	10^2.5 [VDS]	10^2.5	*ND*	29.14	29.57	27.48	5	neg
28	P-GP (trial 1)	in	pig	Conjunctival	swab	P3	6	10^2.0 [VDS]	10^2.0	*ND*	29.07	29.39	27.45	103	neg
29	P-GP (trial 1)	in	pig	leukocytes	wbc	P3	6	10^3.5 [VDS]	neg*	neg*	29.52	29.25	27.28	9	neg
30	P-GP (trial 1)	in	pig	EDTA-blood	blood	P3	6	*ND*	*ND*	*ND*	31.26	ND	27.11	320	neg
31	P-GP (trial 1)	in	pig	Serum	serum	P3	6	*ND*	*ND*		35.58	ND	31.02	360	neg
32	P-GP (trial 1)	cci	pig	Jejunal peyer's patches	tissue	P1	30 (22)	*ND*	*ND*	*ND*	37.26	ND	38.11	-6	neg
33	P-GP (trial 1)	in	pig	Tonsil	tissue	P3	30	*ND*	*ND*	*ND*	33.91	ND	36.10	-12	neg
34	P-GP (trial 1)	in	pig	Bronchial ln.	tissue	P3	30	*ND*	*ND*	*ND*	36.35	ND	36.70	-8	neg
55	W-GP (trial 2)	in	wild boar	EDTA-blood	blood	W1	4	*ND*	*ND*	*ND*	No Cq	ND	32.28	3	neg
56	W-GP (trial 2)	in	wild boar	Leukocytes	wbc	W1	4	10^3.0 [CHS]	10^2.5	10^3.0	No Cq	ND	31.05	-2	neg
57	W-GP (trial 2)	in	wild boar	Serum	serum	W1	4	*ND*	*ND*	*ND*	37.32	ND	36.73	10	neg
58	W-GP (trial 2)	in	wild boar	Fecal	swab	W4	6	10^3.5 [CHS]*	10^3.0*	10^3.5*	29.22	ND	26.50	19	neg
59	W-GP (trial 2)	in	wild boar	Oronasal	swab	W3	7	neg [VDS/CHS]*	neg*	neg*	32.87	ND	30.79	-7	neg
60	W-GP (trial 2)	in	wild boar	Conjunctival	swab	W4	7	neg [VDS/CHS]*	neg*	neg*	32.47	ND	30.04	-7	neg
61	W-GP (trial 2)	in	wild boar	Fecal	swab	W2	10	10^2.5 [VDS/CHS]*	10^2.5*	10^2.5*	29.10	ND	26.06	332	neg
62	W-GP (trial 2)	in	wild boar	Tonsil	tissue	W1	28	*ND*	*ND*	*ND*	31.73	ND	31.09	20	neg

Serial no.	Animal trial ID (trial no.)	Infection status	Animal species	Sample material	Type	Animal ID	dpi (dpci)	SLAM-cells (max. $TCID_{50}$/ml [cell line])	Vero.dog.SLAM .tag ($TCID_{50}$/ml)	CHS-20 ($TCID_{50}$/ml)	Detection of PPRV-Np by RT-qPCR (Cq value) Bao et al. 2008	Polci et al. 2013	Batten et al. 2011	Ag-ELISA (OD NC %)	LFD (pos/ neg)
63	W-GP (trial 2)	in	wild boar	Mediastinal ln.	tissue	W1	28	*ND*	*ND*	*ND*	No Cq	ND	33.03	-10	neg
64	W-GP (trial 2)	in	wild boar	Ileal peyer's patches	tissue	W4	28	*ND*	*ND*	*ND*	No Cq	ND	32.29	-5	neg
65	S-S (trial 4)	in	sheep	EDTA-blood	blood	S3	8	*ND*	*ND*	*ND*	32.38	ND	30.24	127	neg
66	S-S (trial 4)	in	sheep	Serum	serum	S3	8	*ND*	*ND*	*ND*	No Cq	ND	37.81	132	pos
67	S-S (trial 4)	in	sheep	Oronasal	swab	S1	4	10^2.0 [CHS]*	neg*	10^2.0*	No Cq	ND	37.14	-5	neg
68	S-S (trial 4)	in	sheep	Conjunctival	swab	S5	8	10^3.5 [CHS]*	10^2.0*	10^3.5*	26.41	ND	24.03	336	neg
69	S-S (trial 4)	in	sheep	Fecal	swab	S3	8	10^2.0 [VDS/CHS]*	10^2.0*	10^2.0*	26.60	ND	24.09	170	neg
70	S-S (trial 4)	in	sheep	Oronasal	swab	S9	10	10^3.5 [CHS]*	10^2.5*	10^3.5*	25.06	ND	22.37	1	neg
71	S-S (trial 4)	in	sheep	Conjunctival	swab	S7	6	10^2.0 [VDS/CHS]*	10^2.0*	10^2.0*	32.13	ND	27.46	220	neg
72	S-S (trial 4)	in	sheep	Tonsil	tissue	S3	21	*ND*	*ND*	*ND*	33.31	ND	32.17	-5	neg
73	S-S (trial 4)	in	sheep	Mediastinal ln.	tissue	S9	21	*ND*	*ND*	*ND*	32.25	ND	31.31	-5	ND
74	S-S (trial 4)	in	sheep	Ileal peyer's patches	tissue	S7	21	*ND*	*ND*	*ND*	32.02	ND	31.40	-3	ND
	PPRV cell culture virus			Kurdistan/2011	pos control			10^5.5 [CHS]*	10^5.25*	10^5.5*	18.56	ND	ND	ND	pos

dpi, days after experimental infection; dpci, estimated day after contact infection; in, infected by inoculation; inci, intranasally inoculated but infected by contact infection; cci, infected by contact infection; ND, not defined; neg, negative; No Cq, Cq = 45; lgpos, low-grade positive; pos, positive; SLAM cells, cells expressing signaling lymphozyte activation molecule (CD150); VDS, 'Vero.dog.SLAM.tag' vero cells expressing the dog SLAM protein (von Messling et al. 2003); CHS-20, Monkey CV1 cell line expressing the goat SLAM protein (Adombi et al. 2011); LFD, lateral flow device, PESTE-TEST, Field test for Peste des Petits Ruminants Virus Infection, BDSL IRVINE LIMITED and The Pirbright Insitute, Pirbright, UK, detecting PPRV H protein; Ag-ELISA, ID Screen® PPR Antigen Capture sandwich ELISA, ID.vet, detecting PPRV N protein; OD NC %, optical % negative control; wbc, washed white blood cells (leukocytes isolated from EDTA-treated whole-blood by washing procedure); *after freezing and thawing (note: for leukocytes titers may be up to 10^4.5 TCID50/ml lower after freezing-thawing, while for swab samples titers may be up to 10^2.5 TCID50/ml higher after freezing-thawing (data not shown)

Technical Appendix Table 5. Statistical results of Cq values obtained from swab samples collected from different *Artiodactyla* species over time during transmission trials with PPRV lineage IV strain Kurdistan/2011. *P*-values were calculated using i) a linear mixed-effects model (lme) including random effects (individual animal) and fixed effects (animal species and days after infection as continuous variables) (lower left triangle of table) and ii) independent 2-group Mann-Whitney test with Bonferroni correction to adjust the α-level for multiple comparison (upper right triangle of table). All samples significant with the lme model were also significant with the Mann-Whitney U test and vice versa, except for two values of conjunctival swab samples (marked in bold black). In general, goats secreted and excreted statically significantly ($p < 0.05$) higher PPRV-RNA loads over time in all swab materials compared to animals belonging to the other 3 *Artiodactyla* spp. Oronasal secretions were found similarly high between sheep and pigs, but significantly lower for wild boar. Conjunctival secretion patterns were detected significantly different for all species by the Mann-Whitney U test but were similar according to the results of the lme model. For fecal swab samples, PPRV-RNA excretion patterns were found similar for pigs, wild boar and sheep.

**swab material**	**animal spp.** (no.)	**goat** (n = 4)	**pig** (n = 5)	**sheep** (n = 5)	**wild boar** (n = 4)
**oronasal**	**goat**	31.082	0.015	0.013	0.000
	**pig**	0.037	35.244	**0.885**	0.000
	**sheep**	0.021	**0.716**	35.819	0.005
	**wild boar**	0.000	0.011	0.025	39.062
**conjunctival**	**goat**	32.158	0.000	0.014	0.000
	**pig**	0.002	40.303	0.012	0.009
	**sheep**	0.043	**0.122**	37.067	0.000
	**wild boar**	0.000	**0.184**	0.001	42.786
**fecal**	**goat**	31.309	0.000	0.001	0.001
	**pig**	0.002	38.400	**0.388**	**0.978**
	**sheep**	0.005	**0.669**	37.696	**0.614**
	**wild boar**	0.002	**0.898**	**0.576**	38.302

Intercept Cq values for different animal spp. as calculated by lme are highlighted in gray. *P*-values of α <0.05 were considered statistically significant. Non-significant *p*-values are highlighted in **bold** and *p*-values found non-significant in both statistical tests are additionally highlighted in **red**.

Technical Appendix Table 6. Results of spearman nonparametric correlation analyses. Spearman nonparametric correlation analysis of samples collected from the 4 different *Artiodactyla* species pig, wild boar, goat and sheep experimentally infected or contact-infected with small ruminant morbillivirus (PPRV) in transmission experiments. Data of oronasal, conjunctival and fecal swab and of leukocyte samples were combined, but analyzed separately by i) animal species, ii) animal trial, iii) detection method and iv) until versus after seroconversion. A statistically significant correlation between virus titer and Cq value results for samples from pigs (trial 1) collected until seroconversion and from samples of goats (trials 1 and 3) and sheep (trial 4) collected until and after seroconversion, but not for samples from wild boar collected before seroconversion. Similarly, the Spearman correlation coefficient of these data generally varied between a poor (wild boar: rs of −0.02) and fair to good (pigs, goats and sheep: rs between −0.53 and −0.74) inverse correlation. An excellent correlation coefficient (rs of −0.75) was found for samples from i.n. PPRV-infected goats collected until seroconversion. (For visualization of correlation see Appendix Figure 2.)

Animal spp.	Infection status	Serologic status	Trial no.	rs	$CI_{95\%}$ of rs	Interpretation of rs$	*P*-value	No. of samples #
Pig	In, inci	until scv	1	−0.474	−0,604 to −0,320	fair to good	<0.0001*	124
		after scv	1	nd				65
Pig	cc	until scv	3	nd				21
		after scv	3	nd				3
Wild boar	in	until scv	2	−0.016	−0,377 to 0,350	poor	0.9338	31
		after scv	2	nd				5
Goat	in	until scv	3	−0.752	−0,905 to −0,427	excellent	0.0003*	18
		after scv	3	−0.624	−0,886 to −0,059	fair to good	0.0335*	12
Goat	cc	until scv	1	−0.718	−0,810 to −0,592	fair to good	<0.0001*	85
		after scv	1	−0.741	−0,904 to −0,392	fair to good	0.0011*	17
Sheep	in	until scv	4	−0.649	−0,811 to −0,394	fair to good	<0.0001*	35
		after scv	4	−0.533	−0,800 to −0,090	fair to good	0.0188*	19

in, intranasal infection; inci, intranasally inoculated but infected later by contact infection; cc, contact infected; nd, not definable since no vi positive results after seroconversion; scv, seroconversion; rs, Spearman correlation coefficient; CI95%, 95% confidence interval; $, degree of inverse correlation (poor: >-0.75; fair to good: −0.4 to −0.75; excellent: <-0.4); *, significant p-value (α <0.05); #, including samples negative by virus titration

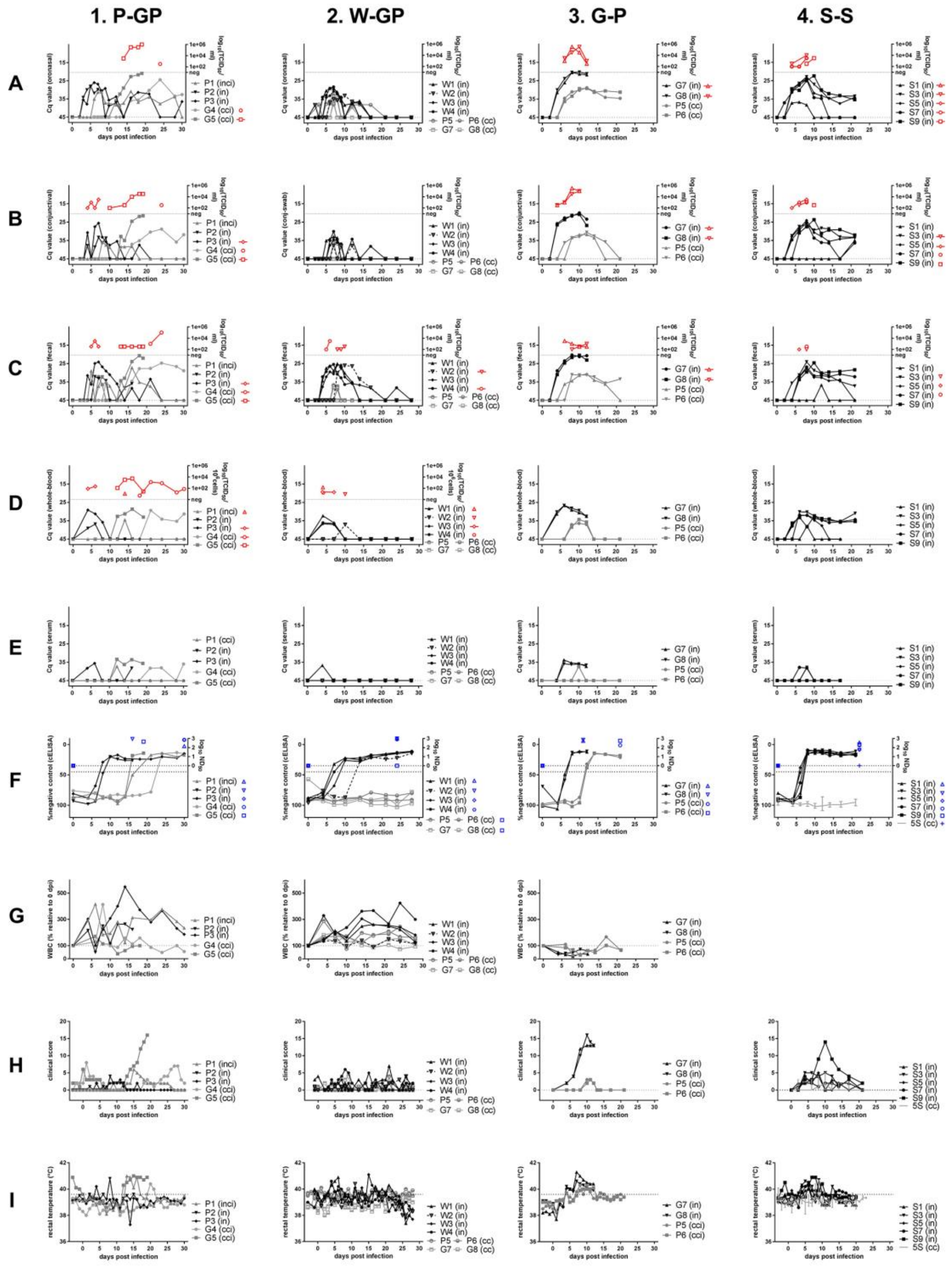

Technical Appendix Figure 1. Virological, serologic, hematological and clinical results and transmission events documented during PPRV-transmission trials after experimental intranasal (in) or contact (cci) infection of pigs (P), wild boar (W), goats (G) and sheep (S) with small ruminant morbillivirus lineage VI peste-des-petits-ruminants virus (PPRV) strain Kurdistan/2011 (*1*,*2*). A-E) RT-qPCR results were determined with thePPRV PCR-assay of Batten et al. (*14*), and virus isolation results were obtained by

using vero.dog.SLAM.tag cells (*4*) and/or CHS-20 (goat-SLAM) cells (*3*) (both cell lines show a similar sensitivity for virus isolation from different animal species, see Appendix Table 3). F) PPRV antibodies were determined with competitive ELISA (cELISA, ID.vet) and by neutralization test. G) Leukocytes (WBC, white blood cell) count was determined relative to 0 dpi. H) The clinical score and I) rectal temperature was documented according to Appendix Table 2, dotted lines determine the threshold of the maximum physiologically normal body temperature. Figure panels have an additional y-axis on the right side, when samples obtained from the respective animal trials were analyzed by virus titration (TCID50/ml) or neutralization test (ND50).

For reasons of clarity, only samples positive by virus isolation are shown in the respective graphs and by animal (red symbols). Furthermore, in the figure legends, red symbols are only displayed for animals that excreted infectious PPRV as measured by virus titration. The presence of neutralizing antibodies (blue symbols) was analyzed in samples taken before experimental or contact infection with PPRV and shortly before or at the day of euthanasia. In the panels 4.F, 4.H and 4.I median and range values of contact control sheep are shown to allow a clearer overview of the data. inci, intranasally inoculated but infected by contact with PPRV; cc, contact control animal that was not infected by contact with PPRV.

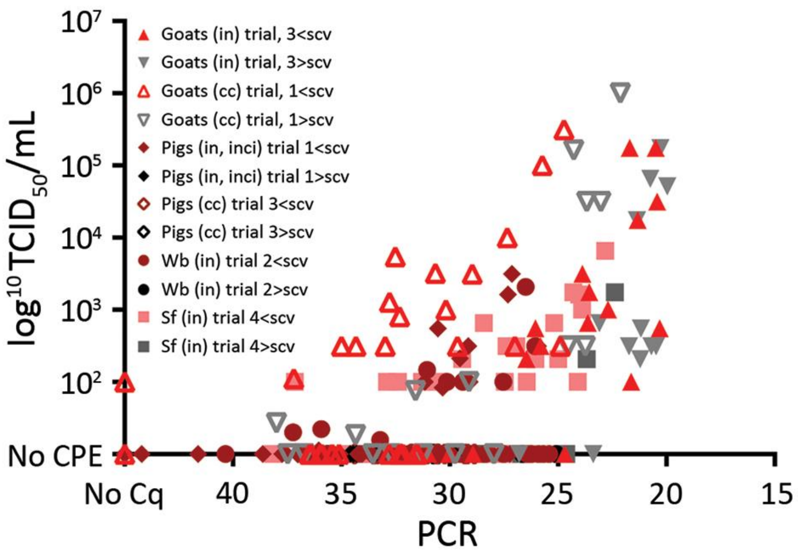

Technical Appendix Figure 2. Correlation of virus titration and RT-qPCR results of oronasal, conjunctival and fecal swab samples and leukocytes from PPRV-infected goats, pigs, wild boar and sheep. PPRV could be isolated from samples with Cq values up to Cq 38 and even from one sample negative by PCR. In general, a high number of samples from goats, pigs, wild boar and sheep with Cq ≤31 were positive by virus titration assay, before (red to pink) or after (gray to black) seroconversion (scv). PPRV could be isolated from all samples with Cq values ≤23. Correlation analysis was conducted with GraphPad Prism 7.02 software (GraphPad Software, Inc., CA, USA). (For the number of analyzed samples and for results of spearman nonparametric correlation analyses see Appendix Table 4.)
